# Evaluating the role of serum uric acid in the risk stratification and therapeutic response of patients with pulmonary arterial hypertension associated with congenital heart disease (PAH-CHD)

**DOI:** 10.3389/fphar.2023.1238581

**Published:** 2023-08-28

**Authors:** Jun Luo, Yuanchang Li, Jingyuan Chen, Haihua Qiu, Wenjie Chen, Xiaoqin Luo, Yusi Chen, Yingjie Tan, Jiang Li

**Affiliations:** Department of Cardiovascular Medicine, The Second Xiangya Hospital, Central South University, Changsha, Hunan, China

**Keywords:** uric acid, congenital heart disease, pulmonary arterial hypertension, eisenmenger syndrome, risk stratification

## Abstract

**Background:** Pulmonary arterial hypertension (PAH) is a malignant pulmonary vascular disease that negatively impacts quality of life, exercise capacity, and mortality. This study sought to investigate the relationship between serum uric acid (UA) level and the disease severity and treatment response of patients with PAH and congenital heart disease (PAH-CHD).

**Methods:** This study included 225 CHD patients and 40 healthy subjects. Serum UA was measured in all patients, and UA levels and haemodynamic parameters were re-evaluated in 20 patients who had received PAH-specific drug treatment for at least 7 ± 1 month.

**Results:** Serum UA levels were significantly higher in PAH-CHD patients than in CHD patients with a normal pulmonary artery pressure and normal subjects (347.7 ± 105.7 μmol/L vs. 278.3 ± 84.6 μmol/L; 347.7 ± 105.7 μmol/L vs. 255.7 ± 44.5 μmol/L, *p* < 0.05). UA levels in the intermediate and high risk groups were significantly higher than those in the low-risk group (365.6 ± 107.8 μmol/L vs. 311.2 ± 82.8 μmol/L; 451.6 ± 117.6 μmol/L vs. 311.2 ± 82.8 μmol/L, *p* < 0.05). Serum UA levels positively correlated with mean pulmonary arterial pressure, WHO functional class, pulmonary vascular resistance, and NT-proBNP (*r* = 0.343, 0.357, 0.406, 0.398; *p* < 0.001), and negatively with mixed venous oxygen saturation (SvO_2_) and arterial oxygen saturation (SaO_2_) (*r* = −0.293, −0.329; *p* < 0.001). UA significantly decreased from 352.7 ± 97.5 to 294.4 ± 56.8 μmol/L (*p* = 0.001) after PAH-specific drug treatment for at least 6 months, along with significant decreases in mean pulmonary arterial pressure and pulmonary vascular resistance and increases in cardiac index and mixed SvO_2_.

**Conclusion:** Serum UA can be used as a practical and economic biomarker for risk stratification and the evaluation of PAH-specific drug treatment effects for patients with PAH-CHD.

## 1 Introduction

Pulmonary hypertension (PH) is a malignant pulmonary vascular disease that is characterized by a progressive increase in pulmonary vascular resistance (PVR) that can eventually lead to right heart failure and death (Galiè et al., 2015). PH is currently sub-classified into five categories. PAH associated with congenital heart disease (PAH-CHD) is the first category of PH, and is primarily characterized by pulmonary arteriole remodeling (Galiè et al., 2015; Simonneau et al., 2019). Nearly 50% of PAH patients have idiopathic, heritable, or drug-induced PAH in Western countries (Simonneau et al., 2019). However, CHD is the most common cause of PAH in China (Jiang and Jing, 2013).

Most CHD patients are diagnosed late for many reasons, at which time the pulmonary vessels have already undergone irreversible remodeling and in some cases Eisenmenger syndrome (ES) is established. ES is not correctable surgically (Dimopoulos et al., 2014). While PAH-specific drugs improve survival (Galiè et al., 2015; Arnott et al., 2018), many patients continually deteriorate. In a Danish nationwide study, Schwartz found that the 1-, 5-, and 10-year mortality rates of PAH-CHD patients were 24%, 44%, and 52%, respectively (Schwartz et al., 2018). Early diagnosis, accurate and convenient disease severity assessment, and the ability to predict changes due to the disease are key prognostic factors for PAH-CHD patients.

The utility of serological biomarkers in PAH has been a recent subject of significant research. Brain natriuretic peptide (BNP), N-terminal pro BNP (NT-proBNP), troponin T (TNT), endothelin-1 (ET-1) and C-reactive protein (CRP) have all been associated with the development of PAH. However, only the measurement of BNP and NT-proBNP are recommended by clinical guidelines and widely used in practice (Nagaya et al., 2000; Leuchte et al., 2007; Foris et al., 2013; Galiè et al., 2015; Pezzuto et al., 2015).

Serum uric acid (UA) is the final product of purine metabolism and is a marker of low cardiac output (CO) and/or tissue hypoxia (Foris et al., 2013; Galiè et al., 2015; Pezzuto et al., 2015). UA plays an important role in the disease evaluation and prognosis of PAH patients with idiopathic PAH and that associated with connective tissue disease (Nagaya et al., 1999; Castillo-Martínez et al., 2016). However, the clinical significance of UA in patients with PAH-CHD has been poorly studied, and quantitative risk stratification of PAH using UA levels has never been attempted. This study aimed to analyze the role of UA in assessing the disease status, risk stratification, and treatment response of PAH-CHD patients to provide the basis for its clinical use.

## 2 Patients and methods

### 2.1 Study population

**FIGURE 1:**
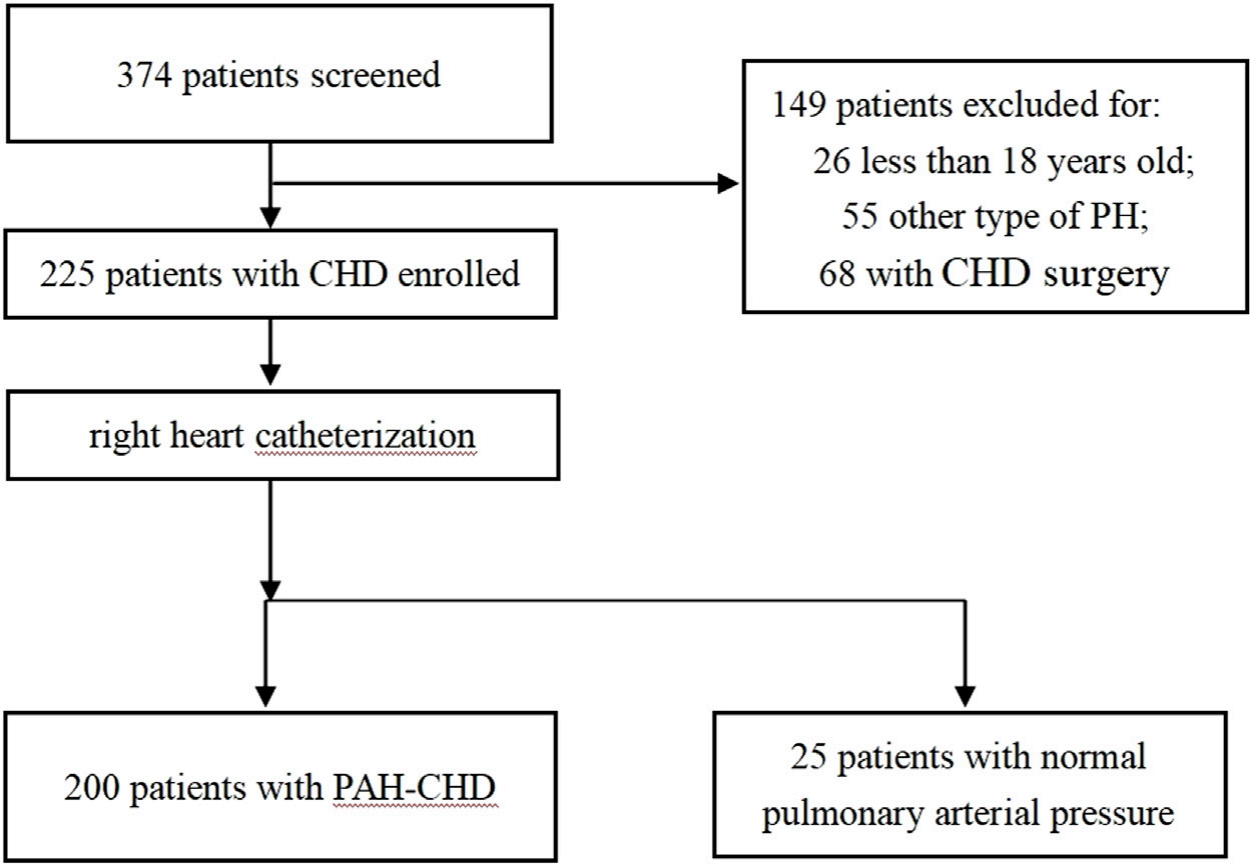
Flow chart of patients selection.

This study was performed at the Department of Cardiovascular Medicine of the Second Xiangya Hospital, Central South University, China. Two-hundred consecutive CHD patients diagnosed with PAH (PAH-CHD group) from July 2017 to June 2019 were enrolled. The inclusion criteria were: 1) a diagnosis of PAH, defined as an mPAP ≥25 mmHg, pulmonary artery wedge pressure (PAWP) ≤15 mmHg, and PVR ≥3 Wood; 2) all measurements were performed via right heart catheterization (RHC). The exclusion criteria were: 1) any other cause of PH, including connective tissue disease, left heart disease, lung disease and/or hypoxia, and chronic thromboembolic disease; 2) patients with gout, liver disease, kidney disease, hypertension, coronary heart disease, an age <18 years old, missing data, a history of CHD surgery, or prior use of PAH-specific drugs. Twenty-five CHD patients with normal pulmonary arterial pressures (non-PAH-CHD group) and 40 healthy adults (normal group) were used as controls. The inclusion criteria for the healthy adult group were age older than 18 years old and no history of hypertension, obesity, diabetes, coronary heart disease, stroke, or other cardiovascular disease. The study protocol was approved by the ethics committee of The Second Xiangya Hospital of Central South University, and all participants provided informed consent before enrollment. The flow chart for this study was described in Figure 1.

### 2.2 Clinical and laboratory data

Baseline clinical information, including gender, age, body mass index (BMI), systolic blood pressure (SBP), diastolic blood pressure (DBP), arterial oxygen saturation (SaO_2_), heart rate, WHO functional class, and previous medical history were collected. Blood samples were drawn from the unilateral cubital vein after an overnight fast and prior to RHC. Medications, including diuretics, cardiac inotropes, and targeted drugs, were initiated based on an adequate risk status assessment. This protocol was implemented to reduce the influence of medication regimen on baseline serum UA level to the greatest extent possible. A complete blood count was analyzed using a SYSMEX Blood Analyzer XN10 (Kobe, Japan). Serum UA levels were measured using the uricase-peroxidase method. Other biochemical parameters, including creatinine (Cr), alanine aminotransferase (ALT), aspartate aminotransferase (AST), direct bilirubin (DBIL), total bilirubin (TBIL), D-Dimer, and NT-proBNP, were analyzed using a Hitachi Automatic Biochemical Analyzer 7,600-020 (Tokyo, Japan) in the laboratory at The Second Xiangya Hospital of Central South University. The reference range for UA was 142.0–416.0 μmol/L.

### 2.3 Echocardiography

An experienced cardiologist performed transthoracic echocardiography (TTE) on all patients using a commercially available device (Vivid 7, Vingmed, GE, United States of America). Using left ventricular (LV) long axis, LV short axis, and apical 4-chamber views, we evaluated and recorded the right atrial end-systolic diameter (RAS), right ventricular end-diastolic diameter (RVD), internal diameter of the pulmonary artery (PA), aortic inner diameter (AO), and left ventricular ejection fraction (LVEF). Systolic tricuspid regurgitation velocity (TRV) was calculated using continuous-wave Doppler echocardiography, and the peak TRV was used to estimate the peak tricuspid regurgitation pressure gradient (PTR) using a simplified Bernoulli equation. Systolic PA pressure (sPAP) was estimated by adding PTR to the estimated right atrial pressure based on the size and change in diameter of the inferior vena cava during respiration.

### 2.4 Right heart catheterization

With the exception of healthy subjects, all patients underwent RHC via their right femoral vein at the Interventional Catheterization Centre of The Second Xiangya Hospital of Central South University. We used a Siemens Axiom Artis X-ray device (SIEMENS Co., Munich, Germany). Baseline haemodynamic parameters, including systolic pulmonary arterial pressure (SPAP), diastolic pulmonary arterial pressure (DPAP), mPAP, mean right atrial pressure (mRAP), PAWP, and oximetry samples of various heart chambers, were measured using a Swan-Ganz catheter. Cardiac index (CI) was calculated as CO based on Fick^’^s principle divided by body surface area. PVR was calculated using the following formula: (mPAP-PAWP)/CO. We used Wood units as the units for PVR. ES was defined as Qp/Qs < 1.0 and PVR >10 Wood. All studied patients were diagnosed with PAH-CHD via RHC according to the following standard criteria: an mPAP≥25 mmHg and PVR>3 Wood units at rest in the presence of a normal PAWP (≤15 mmHg).

### 2.5 Risk assessment

The pre-treatment risk assessment for the 200 PAH-CHD patients was based on the comprehensive risk stratification recommended by the European Heart Journal in 2018 (Kylhammar et al., 2018). This risk assessment strategy includes eight variables: World Health Organization functional class (FC), 6-min walking distance (6MWD), CI, right atrial pressure, NT-proBNP, mixed venous oxygen saturation (SvO_2_), right atrial area, and the presence of a pericardial effusion. All of the aforementioned variables were a part of the risk assessment instrument proposed by the ESC/ERS 2015 guidelines (Galiè et al., 2015). Each variable was categorized as low, intermediate, or high risk based on pre-specified values and given one, two, or three points, respectively. We then divided the sum of all grades by the number of available variables for each patient, resulting in a mean grade. The risk group for each patient was defined by the mean grade rounded off to the nearest integer. PAH-CHD patients were categorized into three risk groups: low (PAH-CHD-L group), intermediate (PAH-CHD-M group), or high (PAH-CHD-H group).

### 2.6 Treatment and follow-up

Surgical indications at our center were a pulmonary-to-systemic flow ratio (Qp/Qs) ≥1.5 and a PVR ≤ 3Wood. The 138 PAH-CHD patients who were not candidates for transcatheter closure or surgery received individualized treatments. In addition to conventional treatment, including oxygen, diuretics, or digitalis, patients with a positive acute pulmonary vasodilator test received a calcium channel blocker. Patients with a negative acute pulmonary vasodilator test received at least one disease-specific drug, such as an endothelin receptor antagonist (ERA), phosphodiesterase type 5 inhibitor (PDE-5i), or prostacyclin analogue (PGI_2_). We followed all PAH-CHD patients who received PAH-specific drug therapies. Twenty PAH-CHD patients underwent repeated RHC after an average follow-up period of 7 ± 1 month. Additional information, including biochemical indicators and echocardiographic parameters, were also collected for these patients.

### 2.7 Statistical analysis

Statistical analyses were performed using the Statistical Package for Social Science version 22.0 for Windows (SPSS Inc., Chicago, IL, United States). Quantitative data (Clinical features, biochemical indicators, echocardiography parameters, and haemodynamic variables) were described as means and standard deviations (normal or approximately normal distributions) or medians and interquartile ranges (IQR) (non-normal distributions). Normally distributed data were compared using a one-way analysis of variance for repeated measurement data, and non-normal distributions were compared using Friedman’s M test. Qualitative data and ranked data (WHO FC) were described as a number and percentage. Ranked data were compared using Friedman’s M test, and qualitative data were compared using Cochran’s Q test. Correlation coefficients between two variables were calculated using Pearson^’^s correlation. A receiver operating characteristic (ROC) curve was used to confirm the serum UA level that provided the best diagnostic significance for intermediate to high-risk PAH-CHD patients. A paired *t*-test was performed to compare baseline and post-treatment echocardiographic, haemodynamic, and serological features. Two-sided *p*-values less than 0.05 were considered statistically significant in all analyses.

## 3 Results

### 3.1 Baseline characteristics

This study included 200 PAH-CHD patients (mean age 37.7 ± 14.1 years; range 15–72 years; 145 female). The PAH-CHD-L group had 94 patients (47.0%), the PAH-CHD-M group 88 (44.0%), and the PAH-CHD-H group 18 (9.0%). All PAH-CHD patients received standard therapy with diuretics. Compared with the nPAH-CHD group and the control group, the body weight and BMI of the PAH-CHD group were lower (*p* < 0.05). There was no statistically significant difference in BMI between the low, medium, and high-risk patients in the PAH-CHD group (*p* > 0.05). There were no significant differences in baseline ALT, AST, total bile acid (TBA), blood Urea Nitrogen (BUN), Cr, international standardized ratio (INR), and D-Dimer levels between groups. Airect bilirubin (DBIL), NT-proBNP, and platelet levels were higher in the PAH-CHD group than in the normal control and nPAH-CHD groups (*p* < 0.05). Subgroup analysis showed that NT-proBNP and platelet levels in the PAH-CHD-M and PAH-CHD-H groups were significantly higher than in the PAH-CHD-L group (*p* < 0.05), but there was no statistical difference between the two groups (*p* > 0.05). The red blood cell distribution width (RDW) in the PAH-CHD group was equivalent to that of the non-PAH-CHD and control groups (*p* > 0.05). However, our subgroup analysis showed that the RDW level of the PAH-CHD-H group was higher than that of the PAH-CHD-M group (*p* < 0.05), but equivalent to the PAH-CHD-L group (Table 1).

**TABLE 1:** Baseline patient characteristics.

Characteristics	Normal (*n* = 40)	Non-PAH-CHD (*n* = 25)	PAH-CHD (*n* = 200)	PAH-CHD-L (*n* = 94)	PAH-CHD-M (*n* = 88)	PAH-CHD-H (*n* = 18)
Clinical features						
Male/Female(n)	14/26	5/20	55/145	19/75	30/58	6/12
Age (years)	41.9 ± 13.1	37.3 ± 12.6	37.7 ± 14.1	35.9 ± 13.9	39.8 ± 14.6	36.4 ± 12.0
BMI (kg/m^2^)	22.8 ± 3.8	21.4 ± 2.9	19.8 ± 3.3^#&^	20.2 ± 3.6^#^	19.4 ± 2.8^#&^	19.4 ± 2.8^#^
HR (beats/min)	81.9 ± 8.4	80.9 ± 13.3	84.4 ± 14.3	81.7 ± 14.2	86.8 ± 14.1^*^	87.2 ± 13.8
SaO_2_ (%)	96.7 ± 1.3	96.3 ± 1.1	92.7 ± 5.5^#&^	93.9 ± 4.0^#&^	93.1 ± 4.4^#&^	84.3 ± 8.8^#&*■^
SBP (mmHg)	117.8 ± 9.1	113.6 ± 11.0	114.4 ± 14.4	114.1 ± 13.1	115.0 ± 15.6	113.1 ± 15.8
DBP (mmHg)	76.2 ± 7.9	74.8 ± 7.2	72.3 ± 10.5	71.6 ± 9.5	72.7 ± 10.7	74.1 ± 14.1
Cardiac diagnosis						
VSD		8	59	16	21	22
ASD		11	68	17	27	24
PDA		6	65	14	25	26
Atrioventricular septal defect			8	1	4	3
Biomarkers						
UA (μmol/L)	255.7 ± 44.5	278.3 ± 84.6	347.7 ± 105.7^#&^	311.2 ± 82.8^#^	365.6 ± 107.8^#&*^	451.6 ± 117.6^#&*^
Cr (μmol/L)	59.6 ± 10.1	60.5 ± 12.9	61.4 ± 15.1	59.1 ± 14.8	64.0 ± 14.8	60.2 ± 17.0
ALT (u/L)	15.6 ± 6.3	14.1 ± 6.7	14.6 ± 6.0	15.2 ± 6.6	14.5 ± 5.6	12.9 ± 4.9
AST (u/L)	18.2 ± 5.7	17.9 ± 4.8	19.4 ± 4.5	19.7 ± 5.3	19.4 ± 3.7	18.4 ± 4.1
TBIL (μmol/L)	10.3 ± 4.5	11.8 ± 3.5	15.6 ± 7.1^#&^	11.7 ± 3.6	17.6 ± 6.9^#&*^	26.4 ± 15.3^#&*^
DBIL (μmol/L)	3.4 ± 1.9	3.8 ± 1.2	5.5 ± 3.8^#&^	4.0 ± 1.4	6.2 ± 3.1^#&*^	9.7 ± 8.5
D-dimer (μg/mL)	0.2 ± 0.1	0.3 ± 0.1	0.3 ± 0.2	0.3 ± 0.2	0.3 ± 0.2	0.4 ± 0.3
NT-proBNP (pg/mL)	78.0 (38.9–121.0)	94.5 (56.8–166.1)	669.8 (213.6–1500.0)^#&^	229.3 (135.3–594.4^)#&^	1155.5 (562.1–2485.8)^#&*^	2301.5 (1464.6-4,672.6)^#&*^
Hb (g/L)	135.0 ± 13.1	129.3 ± 11.2	146.0 ± 26.8^#&^	139.6 ± 23.3^&^	146.5 ± 22.6^#&^	177.3 ± 39.0^#&*■^
PLT (×10^9^/L)	228.1 ± 55.4	217.2 ± 61.6	177.9 ± 52.9^#&^	191.2 ± 50.6^#&^	170.7 ± 51.1^#&*^	143.9 ± 53.9^#&*^
RDW-CV (%)	12.7 ± 0.6	12.8 ± 0.6	14.4 ± 8.6	14.6 ± 12.3	13.6 ± 1.3^#&^	17.3 ± 3.9^#&■^
TTE variables						
RAS (mm)	29.3 ± 2.3	38.0 ± 9.2^#^	42.1 ± 8.8^#^	39.8 ± 6.9^#^	44.7 ± 9.4^#&*^	41.3 ± 11.4^#^
RVD (mm)	29.3 ± 2.1	39.2 ± 9.0^#^	43.3 ± 9.4^#^	41.8 ± 8.6^#^	45.2 ± 9.6^#^	41.9 ± 10.9^#^
LVEF (%)	61.6 ± 3.6	63.1 ± 4.9	62.1 ± 8.1	63.4 ± 7.6	61.7 ± 8.6	57.8 ± 7.3
PA (mm)	20.8 ± 1.6	25.6 ± 6.0^#^	34.7 ± 7.5^#&^	35.1 ± 8.1^#&^	34.9 ± 7.3^#&^	31.3 ± 4.1^#&*^
TRV (m/s)	2.0 ± 0.4	2.7 ± 0.7^#^	4.2 ± 0.9^#&^	4.1 ± 0.9^#&^	4.2 ± 0.9^#&^	4.3 ± 0.9^#&^
Haemodynamic variables						
SPAP (mmHg)	NA	30.2 ± 4.5	89.4 ± 28.7^&^	82.0 ± 27.0^&^	92.6 ± 28.0^&^	112.6 ± 26.4^&*■^
mPAP (mmHg)	NA	20.5 ± 2.7	59.4 ± 21.2^&^	53.8 ± 19.4^&^	61.6 ± 20.4^&^	78.1 ± 22.3^&*■^
mRAP (mmHg)	NA	9.4 ± 3.7	10.9 ± 5.8	9.3 ± 4.1	12.8 ± 6.7^&*^	10.1 ± 6.5
PVR (Wood)	NA	2.3 ± 0.6	11.8 ± 9.8^&^	8.4 ± 5.6^&^	11.4 ± 6.9^&*^	31.9 ± 14.7^&*■^
CI (L/min/m^2^)	NA	3.7 ± 0.8	3.1 ± 0.9^&^	3.2 ± 1.0	2.8 ± 0.6^&*^	1.8 ± 0.4^&*■^
SvO_2_ (%)	NA	75.3 ± 5.7	66.2 ± 8.6^&^	70.4 ± 6.0^&^	64.2 ± 7.8^&*^	53.8 ± 8.3^&*■^

BMI, body mass index; HR, heart rate; SaO_2_, arterial oxygen saturation; SBP, systolic blood pressure; DBP, diastolic blood pressure; UA, uric acid; Cr, creatinine; ALT, alanine aminotransferase; AST, aspartate aminotransferase; TBIL, total bilirubin; DBIL, direct bilirubin; NT-proBNP N-terminal pro-brain natriuretic peptide; Hb, haemoglobin; PLT, platelet; RDW-CV, red blood cell distribution width; RAS, right atrial end-systolic diameter; RVD, right ventricular end-diastolic diameter; LVEF, left ventricular ejection fraction; PA, pulmonary artery; TRV, tricuspid regurgitant velocity; SPAP, systolic pulmonary arterial pressure; mPAP, mean pulmonary arterial pressure; mRAP, mean right atrial pressure; PVR, pulmonary vascular resistance; CI, cardiac index; SvO_2_, venous oxygen saturation; ASD, atrial septal defect; VSD, ventricular septal defect; PDA, patent ductus arteriosus; NA, no data available. ^#^ *p* < 0.05 versus normal subjects; ^&^ *p* < 0.05 versus non-PAH-CHD, group; *p < 0.05 versus PAH-CHD-L, group; ^■^ *p* < 0.05 versus PAH-CHD-M, group.

### 3.2 Hemodynamic data

The mean RAS, RVD, PA, and TRV of the PAH-CHD (including all subgroups) and nPAH-CHD groups were significantly higher than those of the control group (*p* < 0.05). The mean PA and TRV levels in the PAH-CHD group (including all subgroups) were both higher than those of the nPAH-CHD group (*p* < 0.05) (Table 1).

Compared with the nPAH-CHD group, the PAH-CHD group (including all subgroups) had significantly increased pulmonary artery systolic pressure (SPAP), pulmonary artery diastolic pressure (DPAP), pulmonary artery mean pressure (mPAP), right ventricular mean pressure (mRVP), and total pulmonary resistance (PVR) (*p* < 0.05). The levels of SPAP and mPAP in the PAH-CHD-H group were significantly higher than those of the PAH-CHD-M and PAH-CHD-L groups (*p* < 0.05), but there was no statistically significant difference in SPAP between the PAH-CHD-M and PAH-CHD-L groups (*p* > 0.05). The mean DPAP and mRVP of the PAH-CHD-H and PAH-CHD-M groups were higher than those of the PAH-CHD-L group (*p* < 0.05). However, there was no statistically significant difference in DPAP and mRVP between the PAH-CHD-H and the PAH-CHD-M group (*p* > 0.05). SvO_2_ was significantly lower in the PAH-CHD group (including all subgroups), and decreased with increased PAH risk stratification level (*p* < 0.05) (Table 1).

### 3.3 UA levels and ROC curve analysis

**FIGURE 2:**
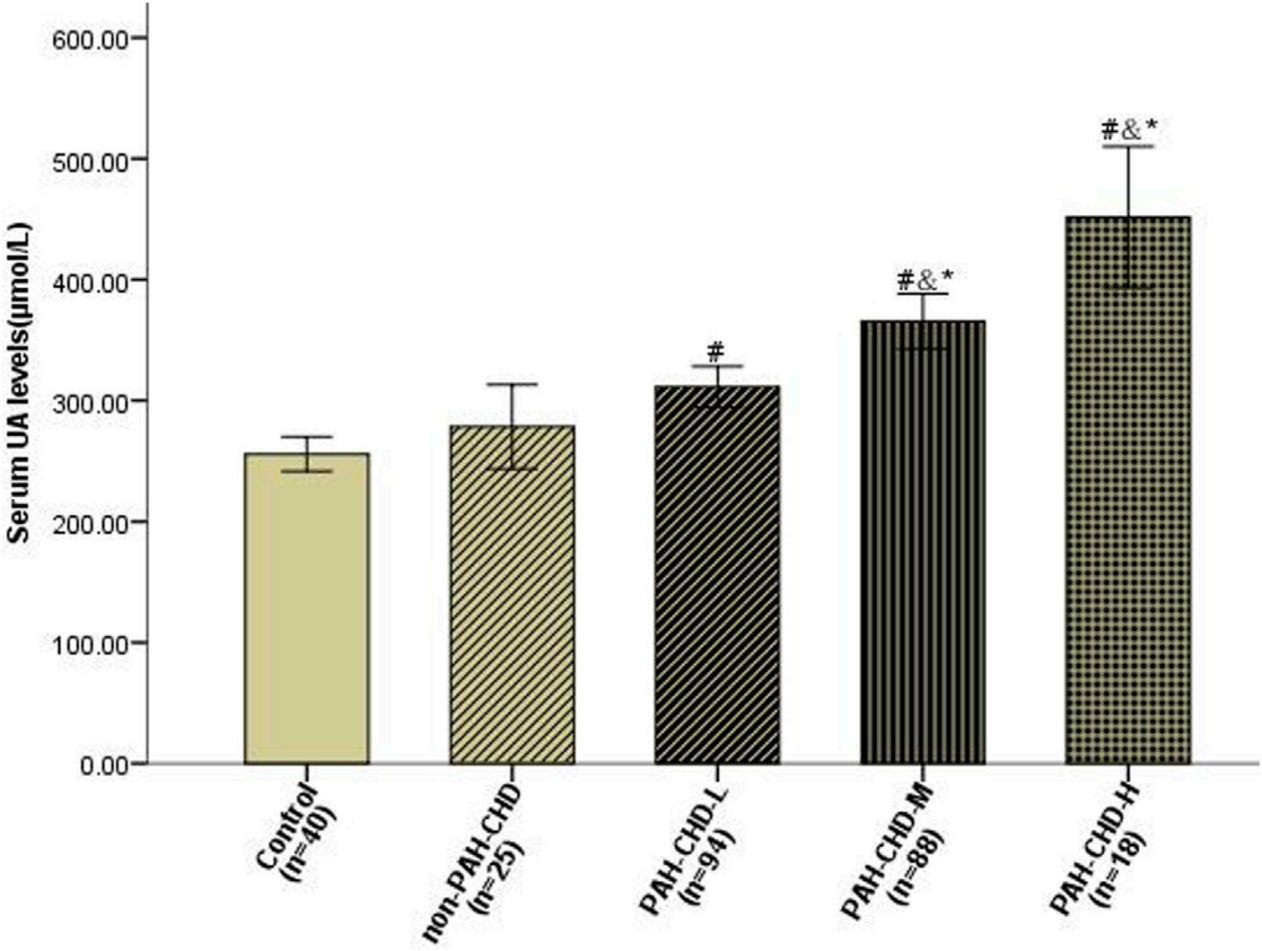
Serum uric acid levels in PAH-CHD patients compared with those in non-PAH-CHD group and normal subjects. ^#^ *P* < 0.05 versus normal control subjects; ^&^ *P* < 0.05 versus non-PAH-CHD group; *P < 0.05 versus PAH-CHD-L group.

**FIGURE 3:**
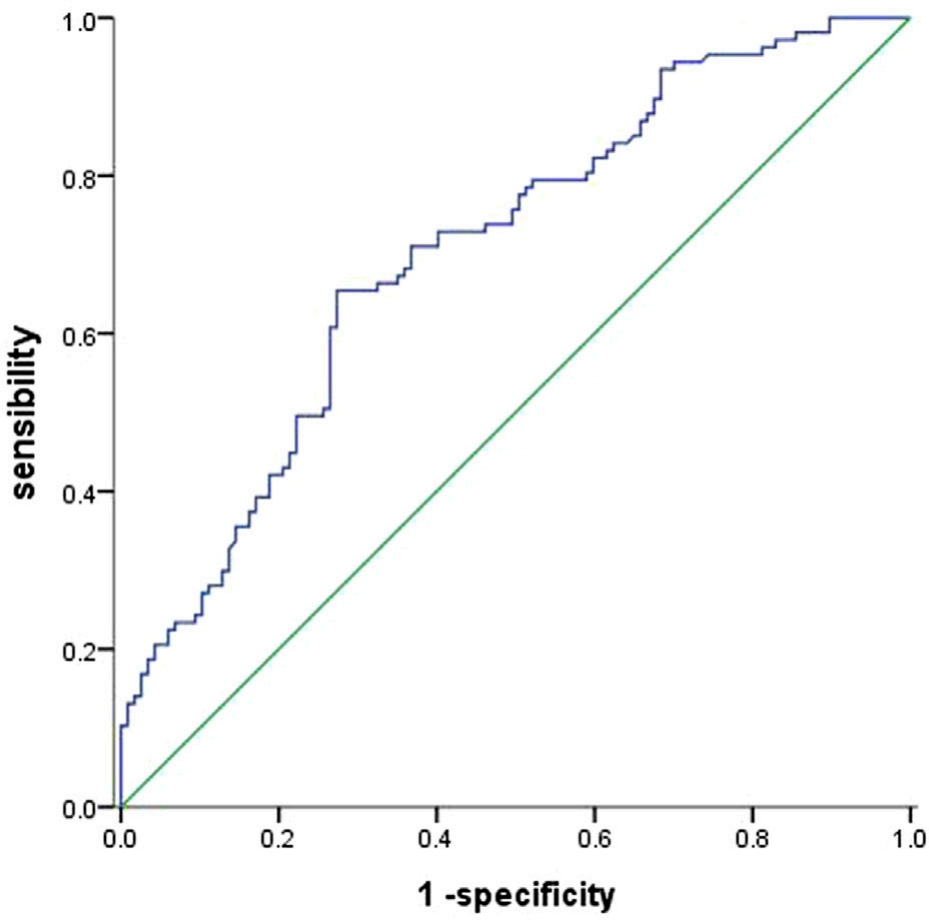
ROC curve evaluating the diagnostic value of serum UA levels for predicting PAH-CHD of intermediate and high risk. An area under the curve (AUC) of 0.706 (95% CI 0.638 −0.773) was obtained with a cut-off point of 330.9 μmol/L.

Baseline UA levels were significantly higher in PAH-CHD patients than in CHD patients with normal pulmonary pressures or normal control subjects (Table 1). Patients in the intermediate- and high-risk groups had significantly higher UA levels than low-risk patients (Figure 2). A total of 40 patients presented with hyperuricemia (UA > 416.0 μmol/L), including 2 non-PAH-CHD patients, 10 PAH-CHD-L patients, 20 PAH-CHD-M patients, and 8 PAH-CHD-H patients, accounting for 8.0%, 10.6%, 22.7%, and 44.4% of each group. As no significant difference in UA levels was observed between the PAH-CHD-L and non-PAH-CHD groups, we performed ROC analysis and identified a cutoff serum UA level of 330.9 μmol/L to achieve the maximum Youden index {sensitivity [65.1%]-[1-specificity (71.4%)]}. The area under the curve (AUC) was 0.706 (95% CI 0.638-0.773) for predicting intermediate-high risk PAH (Figure 3).

### 3.4 Correlations between serum UA levels and haemodynamic features and risk assessment variables

**FIGURE 4:**
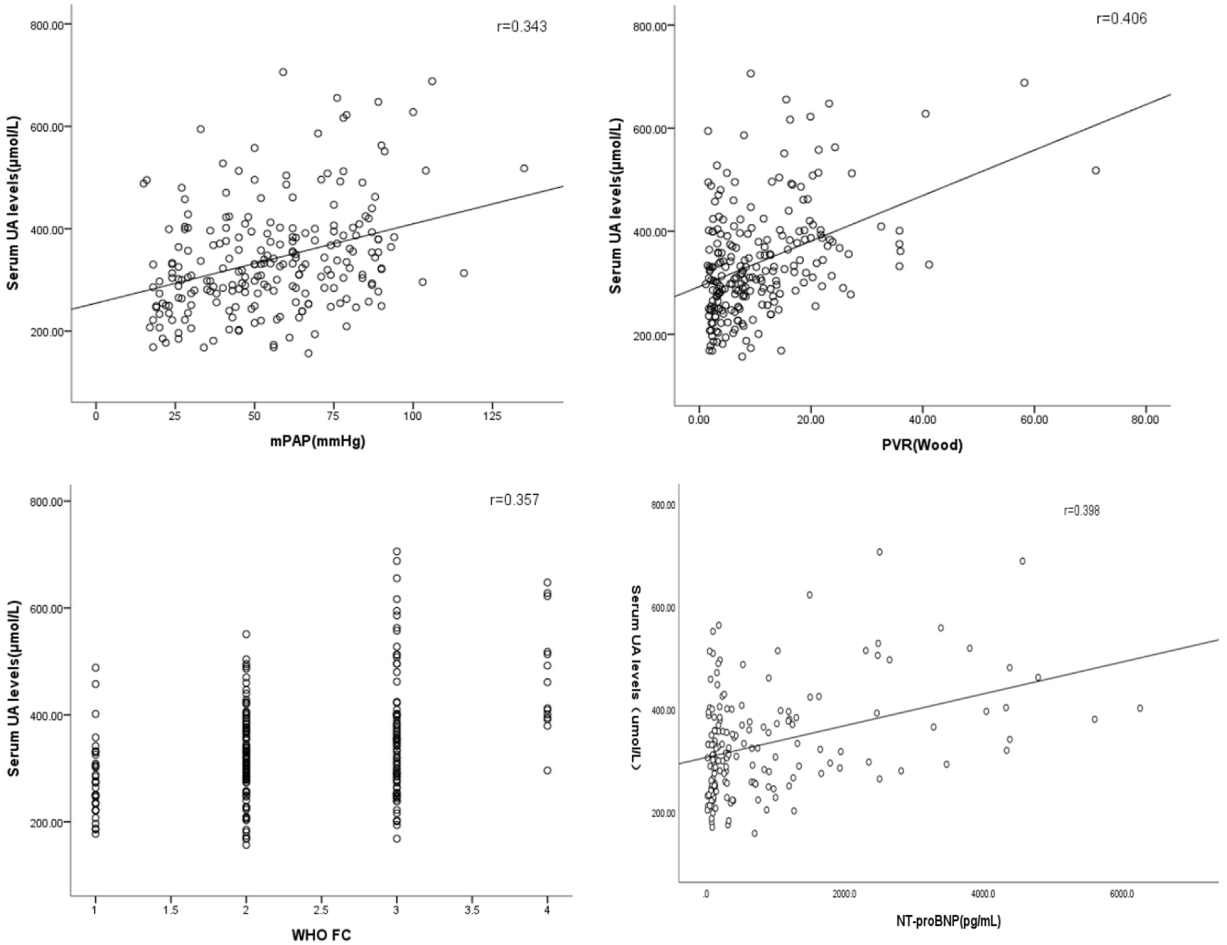
Correlations between serum UA levels, haemodynamic features, and other risk assessment variables.

Table 2 presents correlations between serum UA levels and different variables. Serum UA levels were positively correlated with mPAP, SPAP, WHO functional class, NT-proBNP, and PVR, and a negatively correlated with SvO_2_, and SaO_2_ (Figure 4) the influence of serum UA levels on clinical indicators.

**TABLE 2:** Associations between serum UA levels and other variables.

Variables	Correlation coefficient	95% confidence interval	*p*-Value
SPAP	0.261	0.105 to 0.386	<0.001
mPAP	0.343	0.156 to 0.454	<0.001
mRAP	0.047	−0.105 to 0.260	0.488
PVR	0.406	0.225 to 0.550	<0.001
WHO FC	0.357	0.156 to 0.419	<0.001
NT-proBNP	0.398	0.204 to 0.464	<0.001
CI	−0.183	−0.374 to −0.152	0.006
SvO_2_	−0.293	−0.387 to −0.008	<0.001
SaO_2_	−0.329	−0.411 to −0.127	<0.001

WHO FC, WHO functional class; for others, see Table one.

The optimal cutoff value for UA diagnosis for medium to high-risk PAH-CHD patients was calculated using ROC curve analysis. This study divided PAH-CHD patients into UA > 330.9 μmol and UA ≤ 330.9 μmol groups. Differences in patient characteristics, biochemical indicators, imaging measurements, and hemodynamic parameters were compared between the two groups. Cardiac function grading, NT-proBNP, RAS, RVD, mean PA, and PVR were significantly increased in the higher UA level group than the UA ≤ 330.9 μmol group, while SaO_2_, SvO_2_ and right CI were significantly decreased in the higher UA level group (*p* < 0.05). These results indicate that high levels of UA can indicate more severe PAH and a worse prognosis in PAH-CHD patients (Table 3).

**TABLE 3:** The influence of serum UA levels on clinical indicators.

	UA ≤ 330.9 μmol/L	UA > 330.9 μmol/L	*p*-Value
	(*n* = 122)	(*n* = 103)	
General data			
Age (year)	37.5 ± 12.6	37.8 ± 15.4	0.843
Female (%)	88.5	55.2	<0.001
HR (times/min)	83.8 ± 14.2	84.2 ± 14.2	0.835
^SaO^2^(%)^	94.6 ± 3.6	91.4 ± 6.4	<0.001
cardiac function III-IV(%)	28.7	54.4	<0.001
Biochemical data			
NT-proBNP(pg/mL)	300.0 (132.4-855.9)	977.5 (241.3-2379.8)	<0.001
Echocardiographic parameters			
RAS (mm)	39.5 ± 8.2	44.1 ± 9.2	<0.001
RVD (mm)	40.8 ± 9.0	45.1 ± 9.3	0.001
TRV (m/s)			
LVEF (%)	62.9 ± 7.6	61.4 ± 7.9	0.151
Right Heart Catheter Parameters			
mPAP (mmHg)	49.1 ± 22.4	62.2 ± 22.7	<0.001
mRAP (mmHg)	10.5 ± 5.8	10.9 ± 5.4	0.610
PVR (Wood)	7.5 ± 5.6	14.6 ± 12.0	<0.001
CI(L/min/m^2^)	3.4 ± 0.9	2.9 ± 0.9	<0.001
^SvO^2^(%)^	69.0 ± 7.6	65.1 ± 9.6	0.001

### 3.5 Changes in echocardiographic, haemodynamic, and serologic parameters following vasodilator treatment

All PAH-CHD patients were treated with one or two targeted drugs. Twenty patients followed up for an average of 7 ± 1 month and underwent repeat RHC. During that follow-up period, 10 of the 20 patients received an ERA combined with a PDE-5i, 5 received an ERA with PGI2, 1 received a PDE-5i with PGI2, 1 received PGI2 monotherapy, and the remaining three received ERA monotherapy. After at least 6 months of therapy, serum UA level and other clinical variables were re-examined. Changes in serum UA levels, echocardiographic data, and haemodynamic parameters are presented in Table 4. Haemodynamic and echocardiographic parameters, such as RAS, RVD, sPAP, mPAP, and PVR were significantly decreased, while CI and mixed SvO2 significantly improved after vasodilator treatment. Serum UA levels significantly decreased after vasodilator treatment, from 352.7 ± 97.5 μmol/L to 294.4 ± 56.8 μmol/L (*p* = 0.001). However, no significant decrease in mRAP was observed after treatment.

**TABLE 4:** Changes in clinical parameters due to treatment.

Parameters	Baseline	After therapy	*p*-Value
RAS (mm)	45.7 ± 6.0	41.0 ± 4.4	0.006
RVD (mm)	47.4 ± 7.8	42.4 ± 5.6	0.011
sPAP (mmHg)	91.2 ± 22.1	70.7 ± 19.8	<0.001
SPAP (mmHg)	95.5 ± 20.3	78.9 ± 21.2	<0.001
mPAP (mmHg)	60.3 ± 14.1	50.9 ± 14.8	<0.001
mRAP (mmHg)	9.7 ± 4.1	9.4 ± 3.8	0.786
PVR (Wood)	9.9 ± 6.9	4.5 ± 1.5	0.001
CI (L/min/m^2^)	2.9 ± 0.7	4.5 ± 1.0	<0.001
SvO_2_ (%)	65.2 ± 5.4	69.0 ± 5.6	0.003
UA (μmol/L)	352.7 ± 97.5	294.4 ± 56.8	0.001

sPAP, systolic pulmonary arterial pressure estimated by echocardiography. For other abbreviations see Table 1.

## 4 Discussion

To the best of our knowledge, most of the current research on the role of serum UA levels in PAH has focused on patients with connective tissue diseases and IPAH. The present study found that serum UA levels were significantly elevated in intermediate and high-risk PAH-CHD patients, suggesting that UA is predictive of disease severity. We also found that serum UA levels positively correlated with mPAP, SPAP, WHO functional class, and PVR, and negatively correlated with CI, mixed SvO_2_, and SaO_2_. Serum UA levels also significantly decreased following treatment with PAH-specific drugs. These results suggest that serum UA levels have the potential to serve as an indicator of disease severity and treatment response in PAH-CHD patients.

### 4.1 Increased serum UA levels in PAH-CHD patients

UA levels are be affected by a diverse range of factors, such as gender, age, race, and diet. Hyperuricemia is common in PAH (Nagaya et al., 1999; Voelkel et al., 2000; Dimitroulas et al., 2011). Nagaya et al. (1999) found that increased serum UA levels in idiopathic PAH patients are negatively correlated with CO, and constitute an independent risk factor for long-term mortality. Dimitroulas et al. (2011) reported that systemic sclerosis patients with PAH had higher serum UA levels than those without PAH, and that serum UA levels correlated with 6-min walking and other functional capacity tests. A study conducted on patients with primary and secondary PAH found that serum UA levels were higher in patients with severe PAH (Voelkel et al., 2000).

The results of our research are in agreement with these studies. We found that serum UA levels were significantly higher in patients with PAH-CHD than in non-PAH-CHD patients or healthy subjects. We also noted that hyperuricemia in the present study was mainly present in high-risk PAH-CHD patients.

Although the exact mechanism as to why PAH patients have elevated serum UA levels is unclear, tissue ischaemia and/or hypoxia may play an important role. CHD patients with ES have hypoxic exacerbations due to right-to-left shunting, which may lead to increased serum UA levels (Fathallah and Krasuki, 2018). We hypothesized that the differences in serum UA levels observed within the PAH-CHD group may be due to the higher incidence of ES in the intermediate and high-risk groups. SaO_2_ levels were lowest in PAH-CHD-H patients, and UA levels were negatively correlated with SaO_2_, SvO_2_, and CI, suggesting that uric acid overproduction reflects damage to oxidative metabolism.

Reduced CI and renal perfusion may also be responsible for the elevated serum UA levels. A study by Ross et al. (1986) found that elevated UA levels in ES patients were due to inappropriately low uric acid excretion and enhanced urate reabsorption. Bendayan et al. (2003) reported that hyperuricemia in patients with worsening PAH is caused by impaired renal excretion of UA, which was associated with decreased CO and renal perfusion pressure (Prince et al., 2012). Although serum UA levels were negatively correlated with right CI in the present work, no statistically significant association was found with either serum Cr or LVEF in any of the five groups. These results suggest that patients with normal intrarenal dynamics can appropriately manage elevated UA levels.

Hoeper et al. (1999) found a strong association between serum UA levels and mRAP. This was not the case in our study, although intermediate-risk PAH-CHD patients higher mRAP than CHD patients with normal pulmonary artery pressures. Interestingly, no increases in mRAP were observed in high-risk PAH-CHD patients, which may be explained by right-to-left shunting reducing right atrial pressure.

Diuretic therapy is known to increase serum UA levels by stimulating urate reabsorption in the proximal tubule. All PAH patients received diuretics in our study. However, their baseline renal function was normal, so no significant differences were found between groups. We therefore believe that diuretic use cannot explain the elevated UA level observed in PAH-CHD patients.

### 4.2 Is UA a pathogenic factor in PAH?

While the degree to which elevated UA levels contribute to the development of PAH is unknown, several mechanisms may be proposed to this regard. First, persistent hyperuricemia may result in endothelial dysfunction, which in turn may lead to PAH progression through the promotion of oxidative stress. Besides being a key enzyme for UA production, xanthine oxidoreductase (XOR) is closely related to vascular oxidative stress, plausibly through the generation of reactive oxygen species (ROS) (Berry and Hare, 2004). ROS produced by increased XOR activity may exceeds cellular antioxidant capacity, inducing oxidative stress and endothelial dysfunction (Cai and Harrison, 2000). Second, disruption of NO signaling pathways by UA may be related to the pathobiology of PAH. Pulmonary vascular endothelial cells can induce pulmonary vessel vasodilation by synthesizing NO through the L-arginine-eNOS pathway, a complex process that is catalyzed by eNOS with arginine as a substrate (Zharikov et al., 2008). Third, elevated UA levels stimulate the release of a variety of inflammatory mediators and induce smooth muscle cell proliferation, thereby promoting the development and progression of pulmonary vascular disease (Rao et al., 1991; Kanellis et al., 2003; Kang et al., 2005). The present study lacked a long follow-up time or the use of cardiovascular events as endpoints, but it did find that serum UA levels significantly correlated with prognostic parameters. UA levels may therefore indirectly reflect hemodynamic status, thereby assessing the severity of PAH-CHD.

All 20 patients who received PAH-specific therapy for at least 6 months demonstrated significant improvements in their WHO functional class, echocardiographic parameters, and haemodynamic variables. Serum UA levels decreased and mixed SvO_2_ and right heart CI increased. Oya et al. found that serum UA levels vary in ES patients, with reduced PVR after treatment with PGI_2_ (Oya et al., 2000). These results suggest that serum UA levels may serve as a useful indicator of disease progression and treatment efficacy in PAH-CHD patients.

### 4.3 Targeted therapies in PAH-CHD patients

PAH-CHD is a heterogeneous patient population with various phenotypes of pulmonary vascular disease that range from increased pulmonary blood flow to Eisenmenger physiology with shunt reversal due to supra-systemic pulmonary pressures and right-to-left shunting. There is now additional evidence that PAH-targeted therapies are of benefit to patients with PAH-CHD, and that these therapies are commonly used in this patient population (Rosenkranz et al., 2015; Hidayati et al., 2020; Kaemmerer et al., 2021). This study followed 20 PAH-CHD patients who were re-catherized after at least 6 months of targeted drug treatment. We found that right heart size was significantly improved and hemodynamic indicators, including PVR and PAP, were significantly decreased. This suggests that targeted drug treatment can improve the symptoms and reduce the incidence of cardiovascular events in patients with PAH-CHD. We look forward to a further confirming the role of targeted drugs in congenital heart disease with PH with a larger scale clinical study.

### 4.4 Clinical implications

Serum UA levels are simple to measure in a non-invasive and inexpensive manner. Several additional biochemical markers, including BNP, TNT, ET-1, and CRP, have been proposed, (Nagaya et al., 1999; Leuchte et al., 2007; Castillo-Martínez et al., 2016; Kylhammar et al., 2018), but serum UA may be superior in that it performs as a predictor of the disease severity and mortality of patients with PAH-CHD over long-term follow-up. The present work also found that serum UA levels decreased following PAH-specific drug therapy. Based on these findings, we suggest that serum UA levels be repeatedly measured to evaluate the treatment response of PAH-CHD patients in both the outpatient and inpatient settings.

### 4.5 Limitations

The findings of the present work have to be interpreted in the context of its limitations. First, this is a single center study. Second, although this study had a relatively large sample size compared with related studies in the literature, it was still small for stratified analysis. A larger multi-center prospective study is necessary to further refine our findings. Third, only a few patients underwent repeated RHC after treatment with PAH-specific drugs. The current data can only partially reflect the relationship between decreased UA levels and improved haemodynamic indicators. Fourth, this study lacked a long follow-up time and the use of cardiovascular events as endpoints. We also failed to confirm that whether serum UA levels could act as an independent risk factor for CHD patients with PAH.

### 4.6 Conclusion

In conclusion, we associated serum UA levels with clinical and haemodynamic severity in PAH-CHD patients. Serum UA levels may be a practical biomarker for assessing risk stratification in patients with PAH, and for evaluating treatment response to PAH-specific drugs. Further studies with larger sample sizes are necessary to confirm the results of this study.

## Data Availability

The raw data supporting the conclusion of this article will be made available by the authors, without undue reservation.
